# Chronotype, Lifestyles, and Anthropometric and Biochemical Indices for Cardiovascular Risk Assessment Among Obese Individuals

**DOI:** 10.3390/nu17111858

**Published:** 2025-05-29

**Authors:** Margarida Rabaça Alexandre, Rui Poínhos, CRI-O Group, Bruno M. P. M. Oliveira, Flora Correia

**Affiliations:** 1 Faculty of Nutrition and Food Sciences, University of Porto, 4150-180 Porto, Portugal; ruipoinhos@fcna.up.pt (R.P.); bmpmo@fcna.up.pt (B.M.P.M.O.); 2 Department of Biology and Environment, School of Life and Environmental Sciences, University of Trás-os-Montes e Alto Douro (ECVA, UTAD), Quinta de Prados, 5000-801 Vila Real, Portugal; 3 Centro de Responsabilidade Integrado-Obesidade (CRI-O), Local Health Unit São João, 4200-319 Porto, Portugal; elimadacosta@ulssjoao.min-saude.pt; 4 Laboratory of Artificial Intelligence and Decision Support, Institute for Systems and Computer Engineering, Technology and Science (LIAAD, INESC-TEC), 4200-465 Porto, Portugal; 5 Universidade Lusófona’s Research Center for Biosciences & Health Technologies (CBIOS), Campo Grande 376, 1749-024 Lisboa, Portugal; floracorreia14@gmail.com; 6 Portuguese Society of Nutrition and Food Sciences (SPCNA), 4200-401 Porto, Portugal

**Keywords:** obesity, chronotype, cardiovascular risk, physical exercise, nutrition

## Abstract

Background/Objectives: Obesity is a major contributor to cardiovascular disease, yet traditional risk assessment methods may overlook behavioral and circadian influences that modulate metabolic health. Chronotype, physical activity, sleep quality, eating speed, and breakfast habits have been increasingly associated with cardiometabolic outcomes. This study aims to evaluate the associations between these behavioral factors and both anthropometric and biochemical markers of cardiovascular risk among obese candidates for bariatric surgery. Methods: A cross-sectional study was conducted in a sample of 286 obese adults (78.3% females, mean 44.3 years, SD = 10.8, mean BMI = 42.5 kg/m^2^, SD = 6.2) followed at a central Portuguese hospital. Chronotype (reduced Morningness–Eveningness Questionnaire), sleep quality (Pittsburgh Sleep Quality Index), physical activity (Godin–Shephard Questionnaire), eating speed, and breakfast skipping were assessed. Cardiovascular risk markers included waist-to-hip ratio (WHR), waist-to-height ratio, A Body Shape Index (ABSI), Body Roundness Index, atherogenic index of plasma (AIP), triglyceride–glucose index (TyG), and homeostatic model assessment for insulin resistance (HOMA-IR). Results: Men exhibited significantly higher WHR, ABSI, HOMA-IR, TyG, and AIP. Eveningness was associated with higher insulin (r = −0.168, *p* = 0.006) and HOMA-IR (r = −0.156, *p* = 0.011). Poor sleep quality was associated with higher body fat mass (r = 0.151, *p* = 0.013), total cholesterol (r = 0.169, *p* = 0.005) and LDL cholesterol (r = 0.132, *p* = 0.030). Faster eating speed was associated with a higher waist circumference (r = 0.123, *p* = 0.038) and skeletal muscle mass (r = 0.160, *p* = 0.009). Conclusions: Male sex, evening chronotype, and poor sleep quality were associated with more adverse cardiometabolic profiles in individuals with severe obesity. These findings support the integration of behavioral and circadian factors into cardiovascular risk assessment strategies.

## 1. Introduction

The obesity epidemic remains one of the most challenging public health problems of the 21st century, with its prevalence escalating at an alarming pace across the globe. Beyond impairing quality of life, obesity is a complex metabolic disease, where the excess adiposity is associated with an atherogenic lipid profile, insulin resistance, and systemic inflammation, increasing the risk of cardiovascular disease (CVD) [1]. Unsurprisingly, CVD mirrors obesity’s growing trajectory and is now the leading cause of mortality among individuals with obesity, accounting for nearly 18 million deaths globally and significantly increasing disability-adjusted life-years [2].

Lifestyle interventions, particularly changes in diet and physical activity, remain the foundation of obesity treatment [3]. Physical activity, in particular, is one of the most effective non-pharmacological strategies to reduce cardiometabolic risk by enhancing endothelial function, lipid metabolism, and visceral adiposity reduction. However, many individuals with obesity fail to meet the recommended one hundred and fifty minutes per week of moderate-to-vigorous aerobic physical activity [4].

Beyond these traditional factors, researchers have been recognizing the urge to also consider behavioral and circadian factors as predictors of CVD risk. Among these, chronotype—an individual’s inherent preference for alertness or sleep at specific times of the day [5]—emerges as a key determinant of metabolic regulation. Individuals with an evening chronotype often exhibit higher risk of CVD and mortality because, on the one hand, they have elevated CVD risk factors, such as high triglyceride and C-reactive protein levels, as well as lower high-density lipoprotein cholesterol, and on the other hand, they engage in poorer dietary habits, lower physical activity, and experience misalignment between biological and social clocks [6]. Skipping breakfast is also a common behavior observed in evening individuals, which has also been linked to obesity and arterial stiffness [7,8].

In parallel, poor sleep quality and fast eating speed are increasingly recognized as contributors to obesity and metabolic dysregulation [9,10]. These behaviors impact hormonal secretion, including peptide YY and glucagon-like peptide-1, which regulate satiety and appetite, increasing the risk of obesity and obesity-related cardiometabolic diseases [11]. Sleep disturbances further exacerbate CVD risk by changes in gene expression, enzyme activity, and signaling metabolic pathways [9].

Despite reliance on body mass index (BMI) for obesity diagnosis, BMI alone falls short in distinguishing between lean mass and fat mass or capturing fat distribution [12]. In response, newer anthropometric indicators, including waist-to-hip ratio (WHR), weight-to-height ratio (WHtR), waist-to-height ratio (WHtR), A Body Shape Index (ABSI) [13], and Body Roundness Index (BRI) [14], along with biochemical indices like atherogenic index of plasma (AIP) [15], triglyceride-glucose index (TyG) [16], and homeostatic model assessment for insulin resistance (HOMA-IR) [17], have emerged as robust markers of insulin resistance, subclinical atherosclerosis, and cardiovascular events [18,19,20,21,22], highlighting the value of combining anthropometric and biochemical parameters in cardiovascular risk assessment [23,24]. However, few studies have examined these measures in relation to behavioral patterns such as chronotype, physical activity, sleep, and eating behaviors in a high-risk population of obese candidates for bariatric surgery.

This study aims to address this gap by comparing multiple anthropometric—WHtR, HHtR, WHR, ABSI, and BRI—and biochemical indices—AIP, TyG, and HOMA-IR—as well as body composition measures, including body fat mass and skeletal muscle mass in individuals with severe obesity, candidates for bariatric surgery, accounting for chronotype, skipping breakfast habit, physical activity practice, sleep quality, and eating speed. The primary goal is to determine whether these behavioral traits are associated with distinct cardiometabolic risk profiles, thereby identifying potential predictors of metabolic dysfunction in this high-risk population. We hypothesize that individuals with an evening chronotype, poor sleep quality, fast eating speed, low levels of physical activity, and breakfast skipping habit will exhibit less favorable anthropometric and biochemical profiles, indicative of higher cardiometabolic risk.

## 2. Methods

### 2.1. Study Design, Population, and Sample

This cross-sectional observational study included obese individuals followed in a multidisciplinary assessment for a surgical treatment appointment at the Local Health Unit of São João, Porto, Portugal, between October 2024 and March 2025. All potential participants were candidates for bariatric surgery.

The presence of dependency conditions, namely cognitive impairment, that could constrain free and informed decision-making regarding participation was used as an exclusion criterion, with additional exclusion criteria including having a pacemaker, prosthesis, or any other condition that might affect or be affected by the body composition assessment.

### 2.2. Ethical Considerations

This study was approved by the Ethics Committee for Health of the ULS S. João, University of Porto, in October 2024 (Approval Number 228/2024). It was conducted in accordance with the Declaration of Helsinki for studies in humans [25]. Written informed consent was obtained from all participants prior to enrollment.

### 2.3. Data Collection

#### 2.3.1. Clinical and Anthropometrical Assessment

For all participants, sociodemographic data, including sex, age, marital status, educational level, professional activity, and health-related data, including usual daily medication, health conditions, smoking habits, and food allergies, were collected from the electronic records.

The height was measured using a SECA^®^ 213 portable stadiometer (SECA, Hamburg, Germany) (precision of 0.1 cm). Waist circumference (WC, cm) and hip circumference (HC, cm) were measured using an ergonomic extendable measuring tape SECA^®^ model 201 (precision of 1 mm). All measurements were made following the international standards for anthropometric assessment protocol standardized by the International Society for the Advancement of Kinanthropometry, ISAK [26].

Body composition was assessed through bioelectrical impedance analysis (BIA) using the InBody model 230 (precision of 100 g). Weight (kg), body fat mass (BFM, kg), skeletal muscle mass (SMM, kg), and BMI were obtained during body composition assessment, and the WHO cutoff points for adults were used for BMI classification [27]. BFM and SMM (kg) obtained by BIA were converted to percentage of BFM (% BFM) and percentage of SMM (% SMM).

Based on direct anthropometric assessments, we computed the waist/hip ratio (WHR) by dividing WC by HC, the waist/height ratio (WHtR) by dividing WC by height, and the hip/height ratio (HHtR) by dividing HC by height. Additionally, ABSI [13] was calculated with the following formula:

(1) $$\mathrm{ABSI}=\frac{WC\;(m)}{{BMI\;({kg/m}^{2})}^{2/3}\times {Height\;(m)}^{1/2}},$$

and the BRI [14] was calculated as follows:

(2) $$\mathrm{BRI}=364.2-365.5\times \sqrt{1-\frac{{\left(WC\;(m)/2\pi \right)}^{2}}{{\left(0.5\times height\;(m)\right)}^{2}}}.$$

High cardiovascular risk was classified using the following criteria:

1. WHR: >0.90 male, >0.85 female [28];
2. WHtR: >0.6 [29];
3. ABSI: >0.0801 [30];
4. BRI: >5.9 [31].

#### 2.3.2. Biochemical Assessment

Biochemical data were collected from tests performed within the previous maximum period of 18 months. The blood sample analysis included renal and hepatic function, thyroid function, hormonal profile, vitamins and minerals, lipid profile, namely total cholesterol (TC, mg/dL), high-density lipoprotein cholesterol (HDL-c, mg/dL), low-density lipoprotein cholesterol (LDL-c, mg/dL), and triglycerides (TG, mg/dL), and glycemic profile, including glucose (mg/dL) and insulin (µU/mL).

Additionally, the atherogenic index of plasma (AIP) [15] was calculated:

(3) $$\mathrm{AIP}=\log_{10}\left(\frac{TG\;(mg/dL)}{HD\mathrm{L-c}\;(mg/dL)}\right),$$

as well as the triglyceride–glucose index (TyG) [16]:

(4) $$\mathrm{TyG}=\ln(\frac{TG\;(mg/dL)\times glucose\;(mg/dL)}{2}),$$

and the homeostatic model assessment for insulin resistance (HOMA-IR) [17]:

(5) $$\mathrm{HOMA-}\mathrm{IR}=\frac{fasting\;blood\;glucose\;(mmol/L)\times fasting\;insulin\;(µU/mL)}{22.5}.$$

High cardiovascular risk was classified using the following criteria:

1. AIP: >0.21 [32];
2. TyG > 8.82 male, >8.73 for female [33];
3. HOMA-IR: >3 [34], >10.22 for severe atherosclerosis [35].

#### 2.3.3. Chronotypes

Chronotypes were assessed using an abbreviated 5-item version of the standard 19-item Morningness–Eveningness Questionnaire (MEQ) [36]. The MEQ is one of the most widely used chronotype questionnaires due to its good stability, reliability, coefficient range, and validation in several languages. The reduced MEQ (rMEQ) has emerged as a shorter and simpler way to determine chronotypes, presenting a high correlation (r = 0.898, *p* < 0.00001) with the full form [37].

This five-item questionnaire, validated for the Portuguese population [38], considers sleep and awake times, peak times, morning wakefulness, and self-perceived chronotype, with scores ranging between 4 and 25, where lower scores indicate eveningness and higher scores indicate morningness. The total scores were analyzed both as a quantitative variable and categorized based on standard cutoff scores: morning types (MT, >17), intermediate or neither type (IT, 12 to 17), and evening types (ET, <12) [38].

#### 2.3.4. Physical Activity

The Godin–Shephard Leisure-Time Exercise Questionnaire (GSLTEQ) was used to assess self-reported leisure-time physical activity during a typical 7-day period. It focuses on the frequency of physical activity for bouts of ≥15 min at three intensity levels: strenuous, moderate, and light.

To calculate the leisure-time physical activity score (LTPA), the number of weekly bouts of each activity is multiplied by a corresponding weight to reflect their metabolic equivalent of task (MET) values: LTPA = (9 × Strenuous) + (5 × Moderate) + (3 × Mild) [39].

These weighted values are then summed to produce an overall score. The total scores were analyzed both as a continuous variable or categorized based on standard cutoff scores: insufficiently active/sedentary (<14), moderately active (14 to 23), and active (>24) [40].

#### 2.3.5. Sleep Quality

Sleep quality was assessed using the Pittsburgh Sleep Quality Index (PSQI), validated for the Portuguese population [41]. The questionnaire is composed of 19 questions focusing on sleep quality, latency, duration, efficiency, and disorders, as well as the use of sleep medication and daytime dysfunction, over the previous month. The score may range from 0 to 21, with higher scores indicating poorer sleep quality [42]. PSQI scores were analyzed directly and also categorized into good sleep quality (≤5) or poor sleep quality (>5).

#### 2.3.6. Eating Speed

Eating speed was assessed by asking the following question: “How do you consider your eating speed?”. Participants were asked to classify themselves into “slow”, “moderate”, or “fast” categories, according to their subjective perception.

#### 2.3.7. Skipping Breakfast

Usual breakfast skipping was defined based on the usual waking up time and usual time of first meal. Individuals were considered breakfast skippers if they did not regularly consume any food or beverage (except water) within two hours after waking up.

### 2.4. Statistical Analysis

Statistical analysis was performed using SPSS Statistics software version 29.0, 2023, for MacOS (IBM Company, Chicago, IL, USA).

Normality was assessed using skewness and kurtosis, with quantitative variables being considered to have a normal distribution when both coefficients were between −1 and 1. Quantitative variables that did not follow a normal distribution were transformed using a logarithm function: y = − K + (M + K) × (1 + $\ln(\frac{x+K}{M+K})$), with K > −minimum of x, if skewness was positive, or y = K − (K − M) × (1 + $\ln(\frac{K-x}{K-M})$), with K > maximum of x, if skewness was negative. In our data, K = 21 was considered for the Godin variable and K = 0 for the remaining variables. Descriptive statistics included the means and standard deviations for quantitative variables and relative (%) and absolute (n) frequencies for categorical variables.

Differences between sexes were assessed using chi-square tests (breakfast skipping, chronotypes, physical activity categories, sleep quality categories, and eating speed) or independent-samples *t* tests (anthropometrical and biochemical parameters). The associations with anthropometrical- and biochemical-based variables were measured using Pearson’s (r; rMEQ score, Godin score, and PSQI score) and Spearman’s correlation coefficients (rs; eating speed).

A multivariate analysis (MANCOVA) was performed to study the effects of sex, age, marital status, educational level, shift work, skipping breakfast, chronotype score and categories, physical activity score and categories, sleep quality score and categories, and eating speed on anthropometrical and biochemical indexes. Effect sizes were quantified using partial eta-squared (η_p_^2^) and interpreted as small (η_p_^2^ < 0.030), medium (0.030 ≤ η_p_^2^ < 0.100), or large (η_p_^2^ ≥ 0.100) based on the qualitative definition of between-subject effects originally proposed by Cohen (1988) [43]. A step-by-step approach was used for anthropometric variables, removing the least significant variables for the global model: eating speed (*p* = 0.861), shift work (*p* = 0.702), sleep quality score (*p* = 0.817), sleep quality categories (*p* = 0.678), Godin categories (*p* = 0.790), and Godin score (*p* = 0.546). Similarly, a step-by-step approach was used for biochemical-based variables, removing the less significant variables: educational level (*p* = 0.928), shift work (*p* = 0.809), marital status (*p* = 0.847), eating speed (*p* = 0.641), sleep quality score (*p* = 0.368), and sleep quality categories (*p* = 0.648).

All inferential analysis was performed with 95% confidence. Considering a statistical power of 80%, a correlation of 0.122 is likely to be significant with a sample size of 525 (female subsample size) and a correlation of 0.265 is likely to be significant with a sample size of 109 (male subsample size). Considering a statistical power of 80%, a correlation of 0.165 is likely to be significant with a sample size of 286 participants [44]. In addition to the main analysis, data were also stratified and analyzed by BMI categories: <40 kg/m^2^, [40; 45[ kg/m^2^, [45; 50[ kg/m^2^, and ≥50 kg/m^2^.

## 3. Results

### 3.1. Baseline Characteristics

The sample consisted of 286 patients, of whom 224 (78.3%) were female and 62 (21.7%) were male, with a mean age of 44.3 years (SD = 10.8 years). Most of the sample were married (58.4%) and professionally active (74.1%) individuals. In terms of educational level, the sample was predominantly composed of individuals with middle (33.3%, 6th to 9th grade) and high (38.6%, 10th to 12th grade) education. Regarding smoking habits, 19.6% of our sample were smokers and 22.0% ex-smokers.

Osteoarticular pathology was the most prevalent condition, affecting 50.0% of participants, followed by hypertension (46.9%), dyslipidemia (36.0%), depression (36.0%), obstructive sleep apnea (17.5%), and type 2 diabetes mellitus (14.3%).

#### 3.1.1. Breakfast Skipping, Chronotypes, Physical Activity, Sleep Quality, and Eating Speed by Sex

Baseline sample characteristics regarding breakfast skipping, chronotypes, physical activity level, sleep quality, and eating speed are presented in Table 1.

Males were more likely to skip breakfast (24% vs. 19%, *p* = 0.370) and presented a lower percentage of sedentary behavior (75% vs. 72%, *p* = 0.895), despite non-significant differences between sexes. Regarding chronotypes, most of the sample was classified as IT individuals (46%), followed by MT (33%) and ET (19%), with a similar trend verified between sexes (*p* = 0.324).

Poor sleep quality was observed in 69% of the sample, with males presenting better sleep quality than females (48% vs. 25%, *p* < 0.001). Regarding eating speed, more than half of our sample reported fast eating speed (57%), with a higher proportion among males (75% vs. 52%, *p* = 0.004).

#### 3.1.2. Anthropometric and Biochemical Parameters by Sex

Sex-stratified anthropometrical- and biochemical-based parameters are presented in Table 2.

Males presented a higher mean weight (133.9 vs. 110.2 kg, *p* < 0.001), height (176 vs. 161 cm, *p* < 0.001), SMM (32.7% vs. 27.8%, *p* < 0.001), WC (115 vs. 119 cm, *p* < 0.001), WHR (1.08 vs. 0.90, *p* < 0.001), WHtR (0.77 vs. 0.74, *p* = 0.008), ABSI (0.083 vs. 0.077, *p* < 0.001), and BRI (9.98 vs. 9.08, *p* = 0.007) and a lower mean HC (126 vs. 132 cm, *p* < 0.001), BFM (43.7% vs. 49.9%, *p* < 0.001), and HHtR (0.72 vs. 0.82, *p* < 0.001) compared to females.

Regarding biochemical parameters, females presented a significantly higher mean TC (190 vs. 172 mg/dL, *p* < 0.001), HDL-c (53 vs. 43 mg/dL, *p* < 0.001) and LDL-c (110 vs. 97 mg/dL, *p* = 0.002), whereas males had a significantly higher mean TG (160 vs. 132 mg/dL, *p* = 0.015), fasting glucose (106 vs. 96 mg/dL, *p* < 0.001), insulin (58 vs. 36 µU/mL, *p* = 0.002), AIP (0.52 vs. 0.36, *p* = 0.007), TyG (8.86 vs. 8.62, *p* = 0.007), and HOMA-IR (16.3 vs. 9.9, *p* < 0.001).

When considering the proportion of participants with values indicating high CV risk, males presented higher percentages of values above the defined thresholds compared to females in terms of WHR (98.4% vs. 80.7%), WHtR (100% vs. 98.2%), BRI (100% vs. 96.4%), HOMA-IR > 3 (93.4% vs. 79.2%), HOMA-IR > 10.22 (32.8% vs. 20.8%), ABSI (77.4% vs. 33.6%), AIP (90.2% vs. 75.3%), and TyG (47.5 vs. 40.0%).

### 3.2. Cardiovascular Anthropometric Health Risk Factors

The correlations between rMEQ score, Godin Score, PSQI score, and eating speed and each cardiovascular anthropometric health risk factor (SMM, BFM, WC, HC, WHR, WHtR, HHtR, ABSI, and BRI) are presented in Table 3.

Higher rMEQ scores were associated with lower SMM values (r = −0.126, *p* = 0.040). Higher PSQI scores were associated with lower SMM (r = −0.171, *p* = 0.006), lower WHR (r = −0.159, *p* = 0.008), higher BFM (r = 0.151, *p* = 0.013), and higher HHtR (r = 0.155, *p* = 0.010). Fast eating speed was associated with higher SMM (rs = 0.160, *p* = 0.009) and WC (rs = 0.123, *p* = 0.038). There were no significant associations between Godin Score and anthropometric parameters.

Stratifying by BMI categories, we found similar results across groups, except for the [45; 50[ kg/m^2^ group, where higher rMEQ scores were associated with higher SMM (r = 0.074, *p* = 0.616).

Regarding the PSQI score in the ≥50 kg/m^2^ group, higher scores were associated with higher SMM (r = 0.073, *p* = 0.716). In both the [45; 50[ kg/m^2^ and ≥50 kg/m^2^ groups, higher PSQI scores were associated with lower BFM (r = −0.036, *p* = 0.803 and r = −0.200, *p* = 0.306, respectively).

In terms of eating speed, the <40 kg/m^2^ group exhibited a different pattern, with fast eating speed being associated with lower SMM (*rs* = −0.002, *p* = 0.988). Additionally, in the [40; 45[ kg/m^2^ group, fast eating speed was significantly associated with higher ABSI (*rs* = 0.260, *p* = 0.009).

The multivariate effects of sex, age, marital status, educational level, skipping breakfast, rMEQ score, and chronotypes on each anthropometric characteristic and index are described in Table 4. The model significantly explained all of these features, with medium effect sizes for WHtR and BRI and large effect sizes for all others. Sex had a significant effect on all the dependent variables, with males presenting higher SMM, WC, WHR, WHtR, ABSI, and BRI and lower BFM, HC, and HHtR compared to women.

Also, sex was the characteristic that contributed the most to explaining most of them (with medium to large effect sizes), except for WHtR and BRI, for which education explained a higher proportion of variance (medium effect sizes). Participants with a basic education level had higher WHtR and higher BRI than those with high education.

Participants who skipped breakfast presented lower WHR (medium effect size) and lower ABSI (small effect size). Moreover, the total rMEQ score presented a small but significant effect on SMM, with lower scores (eveningness) associated with higher SMM.

Stratifying by BMI categories, we found similar results across groups, except for the higher BMI groups [45; 50[ kg/m^2^ and ≥50 kg/m^2^. In these categories, individuals with middle educational levels exhibited lower BRI (*β* = −0.237, η_p_^2^ = 0.029, *p* = 0.576 and *β* = −0.426, η_p_^2^ = 0.018, *p* = 0.868, respectively) and WHtR (*β* = −0.009, η_p_^2^ = 0.028, *p* = 0.582 and *β* = −0.018, η_p_^2^ = 0.018, *p* = 0.864, respectively), compared to those with higher education.

Additionally, in the ≥50 kg/m^2^ group, breakfast skippers had a higher ABSI when compared to non-breakfast skippers (*β* = 0.003, η_p_^2^ = 0.028, *p* = 0.510).

### 3.3. Cardiovascular Biochemical-Based Health Risk Factors

The correlations between rMEQ score, Godin Score, PSQI score, and eating speed and each cardiovascular biochemical-based health risk factors (TC, TG, HDL-cm LDL-c, TyG, AIP, glucose, insulin, and HOMA-IR) are presented in Table 5. Higher rMEQ scores were associated with lower insulin (r = −0.168, *p* = 0.006) and HOMA-IR (r = −0.156, *p* = 0.011). Higher PSQI scores were associated with lower TC (r = −0.169, *p* = 0.005), lower HDL-c (r = 0.158, *p* = 0.010), and lower LDL-c (r = 0.132, *p* = 0.030). No significant associations were found between the Godin score, eating speed, and anthropometric parameters.

Stratifying by BMI categories, we found similar results across groups, except for the <40 kg/m^2^ group, where higher rMEQ scores were significantly associated with higher glucose (r = 0.217, *p* = 0.030), while in the other BMI categories, rMEQ scores were non-significantly associated with lower glucose levels. In the same group, fast eating speed was significantly associated with higher HOMA-IR (*rs* = 0.198, *p* = 0.048). Additionally, higher PSQI scores were associated with lower HDL-c in the ≥50 kg/m^2^ group (r = −0.080, *p* = 0.687).

The effects of sex, age, skipping breakfast, chronotype score, chronotype categories, Godin score, and Godin categories on each biochemical-based parameter are described in Table 6. Except for LDL-c, all biochemical parameters were significantly explained by the independent variables in the model, with medium effect sizes for TC and TG, and large effect sizes for HDL-c, TyG, AIP, glucose, insulin, and HOMA-IR. Sex had a significant effect on all the dependent variables, with males presenting lower cholesterol levels (TC, HDL-c, and LDL-c) and higher TG, TyG, AIP, glucose, insulin, and HOMA-IR compared to women. For HDL-c, the effect size of sex was large, whereas for the remaining variables it was medium (TC, LDL-c, TyG, AIP, glucose, insulin, and HOMA-IR) or small (TG).

Age was positively associated with TC (small effect size) and glucose (medium effect size). Glucose was also significantly explained by both rMEQ score and chronotypes (medium effect sizes), with eveningness being associated with lower glucose levels, as well as intermediate type when compared to morning type. rMEQ score was also negatively associated with HOMA-IR (small effect size), with morningness being associated with lower HOMA-IR values.

TG, TyG, and AIP were explained by Godin score (small effect sizes) and physical activity categories (medium effect sizes), with more sedentary participants presenting lower TG, TyG, and AIP. Moreover, Godin categories was the variable that contributed the most to explaining TG and TyG.

Stratifying by BMI categories, we found similar results across groups. However, differences emerged across physical activity categories in individuals with a higher BMI. In the [45; 50[ kg/m^2^ group, sedentary and moderately active individuals exhibited elevated TG levels (*β* = 20.92, η_p_^2^ = 0.001, *p* = 0.869 and *β* = 38.65, η_p_^2^ = 0.005, *p* = 0.656, respectively), when compared to active individuals. In the ≥50 kg/m^2^ group, TG levels were even higher among sedentary and moderately active individuals (*β* = 27.09, η_p_^2^ = 0.002, *p* = 0.849 and *β* = 162.6, η_p_^2^ = 0.125, *p* = 0.137, respectively).

Regarding Tyg, individuals in the ≥50 kg/m^2^ group presented higher values in the sedentary and moderately active categories when compared to active individuals (*β* = 0.399, η_p_^2^ = 0.008, *p* = 0.717 and *β* = 1.089, η_p_^2^ = 0.097, *p* = 0.193, respectively). Similarly, within this BMI group, AIP was higher both in the sedentary group (*β* = 0.222, η_p_^2^ = 0.016, *p* = 0.608) and moderately active group (*β* = 0.550, η_p_^2^ = 0.152, *p* = 0.099) when compared to the active group.

## 4. Discussion

To our knowledge, this is one of the first studies including the interplay between chronotypes, physical activity practice, sleep quality, and eating habits in relation to cardiovascular risk factors in a specific sample of bariatric surgery candidates, while also incorporating newer anthropometric and biochemical indices such as ABSI, BRI, and TyG.

Our main findings were that ET was significantly associated with higher insulin levels and greater insulin resistance, as measured by HOMA-IR. Poor sleep quality correlated with higher body fat mass and a worse lipid profile, with higher total cholesterol and LDL cholesterol. Fast eating speed was associated with increased waist circumference and skeletal muscle mass.

It is important to notice that obesity, by itself, is a major risk factor for CVD risk. However, in Portugal there is a significant prevalence of modifiable risk factors, including hypertension, dyslipidemia, diabetes mellitus, physical inactivity, and tobacco use [45]. In fact, the e_COR study analyzed a sample of 1688 Portuguese individuals and concluded that around 68% of the population presented two or more risk factors for CVD, while 22% had four or more [46]. Aligned with these data, hypertension was presented in 47% of our sample, dyslipidemia in 36%, type 2 diabetes mellitus in 14%, physical inactivity in 73%, and tobacco use in 20%.

Alarming data from 2019 estimated that 30% of all recorded deaths in Portugal were attributed to behavioral risk factors, such as tobacco use, poor diets, alcohol intake, and low levels of physical activity [45]. Given this high-risk profile, it becomes essential to explore which subgroups may present greater cardiovascular risk, accounting not only for sex differences, but also behavioral and individual differences.

Male participants presented higher WC, WHR, ABSI, and BRI and a worse biochemical profile, including higher TG, glucose, insulin, AIP, TyG, and HOMA-IR. On the other side, females presented higher HC, BFM, and HHtR and higher TC, HDL-c, and LDL-c. These results align with sex-specific fat distribution and hormonal patterns. In fact, the conventional view holds that females’ adipose tissue is mainly distributed in the hips and thighs (gynoid obesity), whereas males have an abdominal and upper thoracic distribution of adipose tissue (android obesity) [47]. Besides this, the amount of fat and fat-free mass differs among men and woman; in fact, for the same BMI, woman have a 10% higher fat mass compared to men [48].

Regarding hormonal patterns, estrogen’s regulatory effects contribute to a more favorable lipid profile in women, leading to higher HDL-c and lower LDL-c levels compared to men. However, after 50 years, coinciding with the onset of menopause, women have a steep increase in LDL-c levels, surpassing those observed in men [49]. The Copenhagen General Population Study observed that men have a more atherogenic lipid profile (higher LDL-c and TG levels) in the age groups from 20 to 39 years and 40 to 65 years compared to women in the same age groups [50].

The fact that our sample is majorly composed of obese individuals is a major factor affecting this relationship. In fact, males presented higher TG, although TC and its fractions were higher among females. Our sample’s age distribution may imply that a relevant proportion of female participants were already in the menopause and justify the LDL-C increase in the female subsample. The protective role of endogenous estrogens in women is evidenced by the deleterious impact of the menopause on body composition and glucose homeostasis, with females presenting higher insulin sensitivity and enhanced insulin secretion by pancreatic cells than males [51]. These factors may justify the higher glucose and insulin levels observed in male individuals of our sample, which also had an impact on higher AIP, Tyg, and HOMA-IR values.

The risk factors for CVD show an important sex difference, which may be a contributing factor to the disparity in the prevalence of CVD between sexes. In fact, in Portugal, premature mortality from cardiovascular causes is higher in men, while in women, cardiovascular mortality reaches higher rates starting at age 75 [45].

Skipping breakfast has recently been linked to an increased risk of CVD and all-cause mortality [52]. A cohort study with male U.S. health professionals showed that men who skipped breakfast had a 27% higher risk of coronary heart disease compared with men who ate breakfast [53]. In another large prospective study of U.S adults (40 to 75 years old), after adjusting for confounders, breakfast skippers had an 87% higher risk of dying from cardiovascular disease [54].

In our sample, 20% of individuals were breakfast skippers, with males being more likely to skip breakfast, despite non-significant sex differences. Recent evidence supports these findings, with recent work supporting that high-BMI individuals tend to skip breakfast more than normal-weight individuals, with males presenting a U-shaped relationship between BS and BMI, while for females a J-shaped relationship was found [55].

However, it is important to mention that no direct causal relationship can be drawn from our results or from most of the available data. In fact, skipping breakfast can be linked to stress-independent over-activity in the hypothalamic–pituitary–adrenal axis, higher TC and low HDL-c, and impaired insulin sensitivity, and be by itself a marker of an unhealthy eating pattern and lifestyle. Skipping breakfast is also a factor linked to overweight and obesity development [56].

Skipping breakfast was significantly associated with lower WHR and ABSI in our sample. However, in a systematic review and meta-analysis that included 10 articles, breakfast skipping had no influence on body composition or some obesity indexes, including WHR. It is important to note that the study samples included were small, including participants with a mixed usual breakfast habit who were not exclusively overweight or individuals who skipped breakfast as an attempt to limit their energy intake or reduce their body weight [57].

Regarding chronotypes, higher values on the rMEQ score, corresponding to morningness, were associated with lower SMM, as well as lower insulin and HOMA-IR values. Contrary to our findings, a recent study made among young female individuals concluded that ET presented higher BFM and lower SMM than MT [58]. This discrepancy may be explained by differences in sample characteristics. Our sample consists predominantly of obese, middle-aged individuals, among whom lower SMM may be more prevalent across chronotypes. Furthermore, a significant proportion of our participants (73%) were sedentary, which may contribute to a higher incidence of sarcopenic obesity—characterized by reduced skeletal muscle mass and elevated BFM. Moreover, it should be noticed that a study with a representative sample revealed that the Portuguese population is majorly composed of late-type chronotypes, while almost half of our sample (46%) comprised IT individuals [59].

Relative to glycemic parameters, these results are in line with recent studies evaluating chronotype’s influence on glycemic profile and insulin sensitivity. In fact, IT had lower insulin sensitivity and higher values than MT obese individuals [6]. Another study concluded that ET presented higher insulinemia and HOMA-IR scores, suggesting greater insulin resistance than MT [60]. In our study, eveningness was associated with lower glucose levels, as well as higher HOMA-IR values.

A recent study concluded that ET had higher HOMA-IR values than MT or IT, but no group differences were observed in fasting glucose [61]. However, it is important to notice that an increased HOMA-IR is associated with a higher risk of CVD when compared to fasting glucose or insulin levels. For this reason, despite ET presenting lower fasting glucose, they present higher CVD risk than MT or IT [62].

Regarding sleep quality, in 2018 a Portuguese study with national representativeness found that over 20% of the sample reported short sleep duration (≤5 h) [63]. However, in a more recent study it was found that the average sleep duration of the Portuguese population was 7 h and 53 min, in line with international recommendations [64]. Despite these diverging results, it is widely recognized that obese individuals present worse sleep quality and more sleep disturbances [65]. In fact, our results are in line with such findings, with 69% of our sample presenting bad sleep quality (PSQI > 5), with females presenting higher scores.

Higher PSQI was also associated with lower SMM and WHR, and higher BFM and HHtR. On one hand, the observed differences may be the result of differences in the fat and muscle mass between males and females, with a recent study highlighting this confounder. The same study demonstrated significant associations between all the examined anthropometric and body composition variables and the third PSQI tertile (i.e., the worst sleep quality) in females [66].

Higher PSQI scores were also associated with higher TC, HDL-c, and LDL-c, which is in line with previous studies concluding that short sleep duration is associated with high TC and LDLc [67]. However, the impact of sleep quality on HDL-c is not consensual, with studies reporting diverging directions in the association between poor sleep quality and HDL-c levels [68].

Regarding physical activity practice, it is widely known that it has wide benefits on cardiovascular health, decreasing CVD and mortality. However, a recent cross-sectional study involving 527,662 participants showed that being overweight or obese was associated with increased CVD risk regardless of physical activity level [69], with several studies indicating that high physical activity could attenuate but not eliminate the increased risk of CVD [70]. Most of our sample were sedentary individuals (73%). Surprisingly, lower physical activity was associated with lower TG, TyG, and AIP, contrary to previous studies that highlight the importance of physical activity in decreasing cardiovascular disease risk [71,72]. It is important to mention that sedentary individuals may be the ones that need a more drastic approach and might be polymedicated, resulting in long-term lower lipidic profile values [73].

It has been highlighted that eating speed is strongly associated with BMI, with obese individuals presenting a faster eating speed than normal-weight individuals [74,75]. Indeed, in this study, more than half of the sample reported fast eating speed (57%). Besides this, fast eaters were more likely male individuals than females, results that are in line with recent evidence [76]. In our sample, fast eating speed was associated with higher SMM and WC, which is in line with previous studies [77] and can also be attributed to significant sex differences in eating speed and body composition.

### Strengths and Limitations

This study simultaneously considered anthropometrical indices, such as WHR, WHtR, ABSI, and BRI, and biochemical indices, including AIP, TyG, and HOMA-IR, offering a broad perspective on CVD risk assessment. Besides this, the analysis of individual chronotypes introduces an important feature. In fact, despite growing interest in circadian biology and its influence on metabolism, chronotype remains underrepresented in obesity-related research. By incorporating it into our analysis, we highlight its potential role in shaping health behaviors, such as physical activity patterns and breakfast consumption, as well as its possible physiological effects on lipid metabolism and insulin sensitivity.

Due to study design, casual relationships between chronotype, lifestyle behavior, and metabolic risk cannot be established. Also, physical activity, breakfast consumption, and eating speed were based on single, self-reported items that are prone to recall or social desirability bias, which may distort some of the associations.

The fact that we did not control for the presence of diseases and ongoing pharmacological therapies may have influenced key metabolic indicators, such as lipid levels and insulin resistance markers. This limitation must be taken into account when interpreting our results. Also, the external validity of this study is limited, since all subjects were obese individual candidates for bariatric surgery, which may have introduced a ceiling effect in terms of cardiometabolic risk, potentially masking actual correlations and limiting applicability to non-surgical or less severe individuals. Consequently, the non-significant relationships observed in some analyses may reflect the limited range of phenotypic diversity, rather than the absence of true associations.

Future research including individuals across different BMI ranges, or with varying metabolic profiles, could provide greater clarity.

## 5. Conclusions

This study highlights the relevance of chronotype and behavioral factors—such as sleep quality, physical activity, eating speed, and breakfast skipping—in the assessment of cardiovascular risk among obesity candidates for bariatric surgery.

Beyond traditional anthropometric and biochemical markers, circadian and lifestyle behaviors independently contributed to variations in insulin resistance and lipid-related indices, particularly HOMA-IR and AIP. Male sex, evening chronotype, and poor sleep quality were associated with more adverse cardiometabolic profiles, reinforcing the multifactorial nature of cardiovascular risk in obesity.

These findings support the integration of chronobiological and behavioral assessments into cardiovascular risk stratification models, especially in the high-risk population of obese candidates for bariatric surgery. Future research should explore whether chronotype-aligned interventions may enhance metabolic control and reduce CVD risk in this group.

## Figures and Tables

**Table 1:** Sample characteristics by sex.

	Total (*n* = 286)	Females (*n* = 224)	Males (*n* = 62)	*p* *
	*n* (%)	*n* (%)	*n* (%)	
Breakfast Skippers	57 (20)	42 (19)	15 (24)	0.370
Chronotypes				0.324
MT	95 (33)	73 (33)	22 (36)	
IT	132 (46)	108 (49)	24 (39)	
ET	54 (19)	39 (18)	15 (25)	
Physical Activity				0.895
Active	20 (7)	15 (7)	5 (8)	
Moderately Active	52 (18)	40 (18)	12 (20)	
Sedentary	208 (73)	164 (75)	44 (72)	
Sleep Quality				<0.001
Good Sleep Quality	83 (29)	54 (25)	29 (48)	
Bad Sleep Quality	196 (69)	164 (75)	32 (52)	
Eating Speed				0.004
Slow	25 (9)	23 (10)	2 (3)	
Moderate	98 (34)	85 (38)	13 (21)	
Fast	162 (57)	116 (52)	46 (75)	

* Chi-square test for the independence between sex and each variable. MT: morning type. IT: intermediate type. ET: evening type.

**Table 2:** Anthropometric and biochemical characteristics by sex.

	Total (*n* = 286)	Females (*n* = 224)	Males (*n* = 62)	*p* *
	Mean (SD)	Mean (SD)	Mean (SD)	
Anthropometric				
Weight (kg)	115.1 (21.0)	110.2 (17.5)	133.9 (22.4)	<0.001
Height (cm)	164 (9)	161 (6)	176 (9)	<0.001
BMI (kg/m^2^)	42.5 (6.2)	42.3 (6.1)	43.6 (6.3)	0.284
BFM (%)	48.5 (5.8)	49.9 (4.6)	43.7 (6.8)	<0.001
SMM (%)	33.3 (7.6)	27.8 (2.2)	32.7 (3.5)	<0.001
WC (cm)	122 (15)	119 (13)	135 (14)	<0.001
HC (cm)	130 (13)	132 (12)	126 (12)	<0.001
Waist/hip ratio	0.94 (0.10)	0.90 (0.07)	1.08 (0.08)	<0.001
Waist/height ratio	0.75 (0.08)	0.74 (0.08)	0.77 (0.08)	0.008
Hip/height ratio	0.80 (0.09)	0.82 (0.08)	0.72 (0.07)	<0.001
ABSI	0.079 (0.006)	0.077 (0.006)	0.083 (0.004)	<0.001
BRI	9.28 (2.56)	9.08 (2.59)	9.98 (2.39)	0.007
Biochemical				
Total Cholesterol (mg/dL)	186 (38)	190 (37)	172 (34)	<0.001
HDL-c (mg/dL)	51 (12)	53 (12)	43 (9)	<0.001
LDL-c (mg/dL)	107 (33)	110 (33)	97 (28)	0.002
Triglyceride (mg/dL)	138 (77)	132 (70)	160 (98)	0.015
Glucose (mg/dL)	98 (23)	96 (23)	106 (22)	<0.001
Insulin (µU/mL)	41 (56)	36 (46)	58 (79)	0.002
AIP	0.39 (0.24)	0.36 (0.23)	0.52 (0.25)	0.007
TyG	8.67 (0.57)	8.62 (0.54)	8.86 (0.62)	0.007
Homa-IR	11.3 (20.7)	9.9 (18.5)	16.3 (26.7)	<0.001

* Comparison between sexes (independent-samples Student’s test). SD: standard deviation. BMI: body mass index. BFM: body fat mass. SMM: skeletal muscle mass. WC: waist circumference. HC: hip circumference. ABSI: A Body Shape Index. BRI: Body Roundness Index. HDL-c: high-density lipoprotein cholesterol. LDL-c: low-density lipoprotein cholesterol. AIP: atherogenic index of plasma. TyG: triglyceride–glucose index. HOMA-IR: homeostatic model assessment for insulin resistance.

**Table 3:** Associations between rMEQ score, Godin score, PSQI score, eating speed, and anthropometric variables.

Variables	rMEQ Score		Godin Score		PSQI Score		Eating Speed	
	r	*p*	r	*p*	r	*p*	*rs*	*p*
SMM	−0.126	0.040	−0.032	0.604	−0.171	0.006	0.160	0.009
BFM	−0.029	0.635	−0.048	0.435	0.151	0.013	−0.116	0.055
WC	−0.058	0.337	−0.054	0.366	−0.070	0.247	0.123	0.038
HC	−0.076	0.203	−0.068	0.257	0.086	0.155	0.011	0.854
WHR	0.000	0.999	−0.033	0.581	−0.159	0.008	0.113	0.057
WHtR	−0.008	0.888	−0.034	0.567	0.006	0.921	0.046	0.442
HHtR	−0.012	0.835	−0.035	0.565	0.155	0.010	−0.085	0.154
ABSI	0.079	0.186	0.005	0.940	−0.074	0.219	0.054	0.362
BRI	−0.007	0.910	−0.033	0.581	0.005	0.929	0.046	0.443

r: Pearson’s correlation coefficient. *rs*: Spearman’s correlation coefficient. *p*: significance level (unadjusted for multiple comparisons; considering Bonferroni’s correction, significant correlations if *p*-value < 0.0007). SMM: skeletal muscle mass. BFM: body fat mass. WC: waist circumference. HC: hip circumference. WHR: waist/hip ratio. WHtR: waist/height ratio. HHtR: hip/height ratio. ABSI: A Body Shape Index. BRI: Body Roundness Index. rMEQ: reduced Morningness–Eveningness Questionnaire. PSQI: Pittsburgh Sleep Quality Index.

**Table 4:** Relationships between anthropometrical parameters (SMM, BFM, WC, HC, WHR, WHtR, HHtR, ABSI, and BSI) and sociodemographic, skipping breakfast, and chronotype variables (MANCOVA).

	SMM			BFM			WC			HC			WHR			WHtR			HHtR			ABSI			BRI		
	*β*	η_p_^2^	*p*	*β*	η_p_^2^	*p*	*β*	η_p_^2^	*p*	*β*	η_p_^2^	*p*	*β*	η_p_^2^	*p*	*β*	η_p_^2^	*p*	*β*	η_p_^2^	*p*	*β*	η_p_^2^	*p*	*β*	η_p_^2^	*p*
Corrected model		0.537	<0.001		0.245	<0.001		0.224	<0.001		0.103	0.002		0.523	<0.001		0.087	0.009		0.288	<0.001		0.235	<0.001		0.088	0.009
Sex (Ref.: Female)		0.494	<0.001		0.209	<0.001		0.197	<0.001		0.050	<0.001		0.503	<0.001		0.031	0.005		0.251	<0.001		0.161	<0.001		0.031	0.005
Male	11.00			−6.367			14.95			−6.572			0.175			0.033			−0.105			0.006			0.867		
Age	−0.097	0.031	0.005	−0.036	0.004	0.336	−0.097	0.004	0.291	−0.142	0.010	0.106	0.000	0.001	0.615	0.000	0.000	0.811	0.000	0.002	0.521	0.000	0.011	0.088	−0.004	0.000	0.786
Marital status (Ref.: Other *)		0.003	0.674		0.020	0.077		0.005	0.510		0.012	0.206		0.008	0.352		0.012	0.219		0.018	0.094		0.019	0.083		0.011	0.234
Single	0.946	0.003	0.392	1.876	0.009	0.128	0.670	0.000	0.823	4.243	0.009	0.137	−0.024	0.007	0.168	0.005	0.000	0.786	0.027	0.009	0.137	−0.003	0.019	0.029	0.113	0.000	0.814
Married	0.674	0.002	0.439	−0.109	0.000	0.911	−1.522	0.002	0.519	0.819	0.001	0.716	−0.017	0.006	0.201	−0.015	0.004	0.305	−0.001	0.000	0.942	−0.002	0.014	0.062	−0.388	0.004	0.304
Education (Ref.: High)		0.006	0.458		0.004	0.579		0.016	0.126		0.001	0.868		0.018	0.097		0.047	0.002		0.019	0.089		0.015	0.142		0.048	0.002
Basic	−0.901	0.005	0.284	0.769	0.003	0.411	3.919	0.012	0.086	0.848	0.001	0.695	0.025	0.014	0.062	0.045	0.040	0.001	0.029	0.017	0.036	0.002	0.015	0.053	1.206	0.042	0.001
Middle	−0.930	0.005	0.242	0.010	0.000	0.991	0.487	0.000	0.820	−0.074	0.000	0.971	0.004	0.000	0.744	0.012	0.004	0.336	0.010	0.002	0.429	0.001	0.003	0.368	0.348	0.004	0.311
Skipping breakfast (Ref.: No)		0.006	0.213		0.007	0.178		0.008	0.149		0.003	0.407		0.032	0.004		0.006	0.226		0.004	0.287		0.017	0.037		0.006	0.235
Yes	−0.949			1.140			−2.970			1.626			−0.035			−0.015			0.013			−0.002			−0.391		
Chronotype score	−0.387	0.017	0.036	0.167	0.003	0.414	0.667	0.007	0.181	−0.582	0.006	0.221	−0.001	0.001	0.637	−0.004	0.007	0.179	−0.004	0.006	0.237	0.000	0.001	0.628	−0.106	0.007	0.184
Chronotypes (Ref.: MT)		0.018	0.097		0.003	0.661		0.005	0.538		0.008	0.349		0.003	0.713		0.004	0.595		0.008	0.342		0.002	0.739		0.004	0.604
IT	−2.048	0.016	0.045	0.575	0.001	0.612	−2.837	0.004	0.303	−2.286	0.003	0.384	−0.007	0.001	0.678	−0.013	0.003	0.419	−0.010	0.001	0.557	0.001	0.001	0.541	−0.362	0.003	0.412
ET	−4.378	0.017	0.035	1.956	0.003	0.397	−6.082	0.005	0.278	−7.368	0.007	0.169	0.002	0.000	0.948	−0.034	0.004	0.310	−0.042	0.006	0.212	0.002	0.002	0.438	−0.901	0.004	0.315

SMM: skeletal muscle mass. BFM: body fat mass. WC: waist circumference. HC: hip circumference. WHR: waist/hip ratio. WHtR: waist/height ratio. HHtR: hip/height ratio. ABSI: A Body Shape Index. BRI: Body Roundness Index. *β*: standardized regression coefficient for parameter estimates. η_p_^2^: partial eta-squared. *p*: significance level. * Divorced + Widow(er). MT: morning type. IT: intermediate type. ET: evening type.

**Table 5:** Associations between rMEQ score, Godin score, PSQI score, eating speed, and biochemical-based variables.

Variables	rMEQ Score		Godin Score		PSQI Score		Eating Speed	
	r	*p*	r	*p*	r	*p*	*rs*	*p*
TC	−0.038	0.530	−0.036	0.552	0.169	0.005	−0.023	0.701
TG	−0.080	0.190	0.051	0.406	0.007	0.913	0.085	0.159
HDL-c	0.060	0.324	0.020	0.737	0.158	0.010	−0.034	0.574
LDL-c	−0.029	0.629	−0.072	0.240	0.132	0.030	−0.066	0.279
TyG	−0.078	0.197	0.046	0.452	0.010	0.874	0.074	0.224
AIP	−0.095	0.120	0.021	0.725	−0.054	0.376	0.085	0.160
Glucose	−0.022	0.722	0.022	0.720	−0.027	0.659	0.010	0.864
Insulin	−0.168	0.006	0.112	0.066	−0.079	0.197	0.076	0.210
HOMA-IR	−0.156	0.011	0.109	0.076	−0.074	0.230	0.081	0.184

r: Pearson’s correlation coefficient. *rs*: Spearman’s correlation coefficient. *p*: significance level (unadjusted for multiple comparisons; considering Bonferroni’s correction, significant correlations if *p*-value < 0.0007). TC: total cholesterol. TG: triglyceride. HDL-c: high-density lipoprotein cholesterol. LDL-c: low-density lipoprotein cholesterol. TyG: triglyceride–glucose index. AIP: atherogenic index of plasma. HOMA-IR: homeostatic model assessment for insulin resistance. rMEQ: reduced Morningness–Eveningness Questionnaire. PSQI: Pittsburgh Sleep Quality Index.

**Table 6:** Relationships between biochemical parameters (SMM, BFM, WC, HC, WHR, WHtR, HHtR, ABSI, and BSI) and sociodemographic, skipping breakfast, and chronotype variables (MANCOVA).

	TC			TG			HDL-c			LDL-c			TyG			AIP			Glucose			Insulin			HOMA-IR		
	*β*	η_p_^2^	*p*	*β*	η_p_^2^	*p*	*β*	η_p_^2^	*p*	*β*	η_p_^2^	*p*	*β*	η_p_^2^	*p*	*β*	η_p_^2^	*p*	*β*	η_p_^2^	*p*	*β*	η_p_^2^	*p*	*β*	η_p_^2^	*p*
Corrected model		0.073	0.020		0.072	0.022		0.183	<0.001		0.062	0.053		0.105	<0.001		0.129	<0.001		0.137	<0.001		0.109	<0.001		0.107	<0.001
Sex (Ref.: Female)		0.040	0.001		0.022	0.016		0.124	<0.001		0.030	0.005		0.035	0.002		0.075	<0.001		0.052	<0.001		0.037	0.002		0.047	<0.001
Male	−17.96			27.39			−9.874			−13.573			0.250			0.155			8.913			9.057			2.680		
Age	0.525	0.021	0.021	−0.889	0.000	0.821	0.202	0.014	0.160	0.302	0.003	0.647	0.007	0.007	0.422	0.000	0.004	0.597	0.385	0.032	0.016	−0.232	0.005	0.543	−0.029	0.010	0.281
Skipping breakfast (Ref.: No)		0.000	0.897		0.001	0.677		0.010	0.103		0.001	0.583		0.000	0.767		0.002	0.527		0.002	0.467		0.005	0.276		0.005	0.274
Yes	−0.779			−5.180			−2.946			3.203			−0.027			0.024			−1.910			−3.454			−0.908		
Chronotype score	−0.402	0.000	0.784	−0.889	0.000	0.768	0.706	0.010	0.107	−0.929	0.002	0.467	−0.041	0.014	0.057	−0.012	0.007	0.182	−2.080	0.040	0.001	−1.507	0.015	0.050 *	−0.492	0.023	0.015
Chronotypes (Ref.: MT)		0.004	0.591		0.000	0.981		0.014	0.160		0.003	0.647		0.007	0.422		0.004	0.597		0.032	0.016		0.005	0.543		0.010	0.281
IT	7.255	0.003	0.371	3.013	0.000	0.857	4.263	0.012	0.079	2.390	0.000	0.735	−0.123	0.004	0.307	−0.043	0.003	0.395	−8.604	0.023	0.015	−4.067	0.004	0.339	−1.513	0.007	0.175
ET	7.391	0.001	0.657	6.675	0.000	0.846	9.326	0.014	0.061	−3.270	0.000	0.822	−0.323	0.007	0.190	−0.105	0.004	0.310	−20.97	0.031	0.004	−9.613	0.005	0.272	−3.642	0.010	0.112
Godin score	−0.225	0.001	0.662	−2.561	0.022	0.016	0.055	0.000	0.722	0.232	0.001	0.605	−0.019	0.024	0.012	−0.009	0.027	0.008	−0.085	0.001	0.704	−0.073	0.000	0.786	−0.022	0.000	0.750
Godin categories (Ref.: Active)		0.001	0.850		0.039	0.006		0.005	0.598		0.009	0.306		0.041	0.005		0.039	0.006		0.004	0.604		0.006	0.439		0.006	0.455
Moderately active	−6.942	0.001	0.570	−33.59	0.007	0.183	3.257	0.003	0.372	−3.481	0.000	0.744	−0.337	0.013	0.063	−0.138	0.013	0.069	−5.303	0.004	0.319	−2.559	0.001	0.690	−0.988	0.001	0.556
Sedentary	−9.106	0.001	0.616	−103.1	0.029	0.006	2.592	0.001	0.633	8.926	0.001	0.574	−0.825	0.035	0.002	−0.338	0.034	0.003	−7.092	0.003	0.370	−9.612	0.004	0.314	−2.758	0.005	0.270

TC: total cholesterol. TG: triglyceride. HDL-c: high-density lipoprotein cholesterol. LDL-c: low-density lipoprotein cholesterol. TyG: triglyceride glucose index. AIP: atherogenic index of plasma. HOMA-IR: homeostatic model assessment for insulin resistance. *β*: standardized regression coefficient for parameter estimates. η_p_^2^: partial eta-squared. *p*: significance level. MT: morning type. IT: intermediate type. ET: evening type. * *p* > 0.05.

## Data Availability

Data supporting the findings of this study are available upon request from the corresponding author, subject to ethical considerations.
